# Long-Term Sonographical Follow-Up of Arterial Stenosis Due to Spontaneous Cervical Artery Dissection

**DOI:** 10.3389/fneur.2021.792321

**Published:** 2022-02-03

**Authors:** Daniel Strunk, Wolfram Schwindt, Heinz Wiendl, Ralf Dittrich, Jens Minnerup

**Affiliations:** ^1^Department of Neurology With Institute of Translational Neurology, University Hospital Münster, Münster, Germany; ^2^Clinic of Radiology, Section of Interventional Neuroradiology, University Hospital Münster, Münster, Germany; ^3^Department of Neurology, Marienhospital Osnabrück, Osnabrück, Germany

**Keywords:** spontaneous cervical artery dissection, neurovascular ultrasound, stenosis, stroke, rare causes of stroke

## Abstract

**Purpose:**

Little is known about the long-term course of arterial stenosis after spontaneous cervical artery dissection (sCAD). We analyzed changes over time and evaluated factors potentially associated with these changes and recurring sCAD.

**Materials and Methods:**

Adult patients with sCAD, admitted to our neurological department between 2004 and 2018, were included. All patients underwent initial and follow-up repetitive neurovascular ultrasound for a mean duration of 15.3 ± 21 months. Clinical and imaging data were registered for each patient.

**Results:**

A total of 259 sCADs were diagnosed in 224 patients. Either internal carotid arteries (*n* = 133, 59.4%), vertebral arteries (*n* = 58, 25.9%), or multiple arteries (*n* = 33, 14.7%) were affected. In 93 out of 183 patients (51%), and in 117 out of 210 arteries under investigation (55.7%), vascular stenosis decreased over time. Occluded arteries recanalized early in 34 (54%) and stayed occluded in 29 patients (46.0%). Of 145 initially hemodynamically relevant stenosis, 77 (53.1%) improved over time. Overall, 12 patients (5.4 %) had a recurring sCAD during follow-up. Pseudoaneurysms were found in 19 patients.

**Conclusion:**

The sonographical course of sCAD is highly dynamic within the first year after disease onset and should be monitored carefully. Decreasing degrees of stenosis and recanalization of occluded arteries occurred in half of all patients. Recurrent sCAD was a rare event in our cohort.

## Introduction

In spontaneous cervical artery dissection (sCAD), the outer arterial wall is primarily affected. The pathophysiological mechanism of sCAD is the formation of an intramural hematoma along the medial/adventitial border due to rupture of vasa vasorum and neoangiogenetic capillaries (1). Disintegration of the anatomical structures leads to intimal damage and facilitates thrombus formation. sCAD is therefore a frequent cause of embolic stroke in younger adults, especially in the age group of 20–40 years (2). The intramural hematoma can be visualized by neurovascular ultrasound as an echolucent wall thickening leading to a non-atherosclerotic stenosis.

Previous studies showed a tendency of decreasing degrees of stenosis caused by sCAD, primarily in the first 6 months after disease onset (3–5). Other investigators reported some degree of recanalization in 58.8% of patients, being more frequent in women, in a larger restrospective case series (*n* = 177) (6). The average time to total or near-total recanalization was 4.7+/−2.5 months. Completely occluded arteries at presentation tended not to recanalize (6). It is noteworthy that cases of traumatic CAD were included in the analysis as well. Arauz et al. (7) (*n* = 130) found better clinical outcomes in sCAD of the vertebral artery (VA) compared to cases of affected internal carotid artery (ICA), particularly in patients with demonstrated complete recanalization, in a Mexican cohort.

In this study, we sought to find the long-term course of arterial stenosis after sCAD in a large cohort with long follow-up period by repetitive neurovascular ultrasound. We analyzed the characteristics of patients associated with the long-term course of arterial stenosis and sCAD recurrence.

## Materials and Methods

We conducted a retrospective analysis of patients admitted between 2004 and 2018 to the Department of Neurology, Institute of Translational Neurology, at the University Hospital of Münster, a tertiary care center. Eligible patients were identified by searching the database for the following ICD-10-Codes (ICD = International Classification of Diseases by the World Health Organization): I63.8, I67.0, I72.5, I72.6, I72.0. Our search yielded 259 sCADs in 224 patients.

At our department, computed tomography angiography (CTA) or, preferentially, magnetic resonance imaging (MRI) and angiography (MRA) is an essential part of the initial management of patients with clinically suspected sCAD. Clinical suspicion was based on one or a combination of the following symptoms and diagnoses: neck pain, headache, Horner's syndrome, and stroke in patients of young age. Characteristic radiological features of sCAD are (1) smooth or slightly irregular tapered stenosis (detectable by CT and MR); (2) arterial Occlusion (Rattail-shaped, tapered, flame-like) (detectable by CT and MR); (3) lumen irregularity, irregular dilatation (detectable by CT and MR); (4) pseudoaneurysm (saccular or fusiform aneurysmal dilatation) (detectable by CT and MR); (5) intimal flap (detectable by CT and MR); (6) wall thickening (e.g., suboccipital rind sign) (detectable only by CT); (7) crescent sign (methemoglobin of the intramural hematoma in axial T1-weighted fat-suppressed images) (detectable only by MR) (8).

A neurosonographical examination was carried out in all patients within 48 h after hospital admission. All patients underwent initial and follow-up repetitive neurovascular ultrasound (mean number of total ultrasound examinations: 2.75) for a mean duration of 15.3 ± 21, 27.2 ± 20.7 months in the subgroup with the longest follow-up period.

A total of 84 patients with clinically suspected sCAD were examined using different MRI machines over time. Imaging protocol consisted of 1.5 or 3 Tesla contrast-enhanced MRA, T1-weighted, fat-suppressed axial sequences of the cervical soft tissues, T2-weighted, axial sequences of the region of interest, and in many cases, black blood imaging (9). Main focuses of image analysis were presence and size of intramural hematoma, degree of stenosis, presence of intraluminal thrombus, pseudoaneurysm, and further dissections.

In 91 patients, CT/CTA scans were performed on 128-slice dual source CT scanners (Siemens Somatom Definition AS and Definition Flash; Siemens Medical Solutions, Forchheim, Germany). Non-contrast head images were obtained from the vertex to the skull base (120 kV, 340 mAs, 5.0 mm slice reconstruction, 1.0 mm increment, 0.6 mm collimation, 0.8 pitch, and H30s soft kernel). Afterwards, CTA was performed (120 kV, 175 mAs, 1.0 mm slice reconstruction, 1 mm increment, 0.6 mm collimation, 0.8 pitch, H20f soft kernel, 80 mL Ultravist 370, and 50 ml NaCl flush at 4 ml/s, scan starts 6 s after bolus tracking at the level of the ascending aorta). The scanning was triggered by a CT technologist on the basis of contrast enhancement in the aortic arch following administration of 80 ml of Ultravist with a concentration of 350 mg I/ml (Bayer, Leverkusen, Germany), at a rate of 4–5 ml/s. The CT angiographic source images were post-processed to create coronal and sagittal reformatted images with a 1 mm section thickness, maximum intensity projection (MIP) images, and curved planar reformatted images of the bilateral common and internal carotid and vertebral arteries (10).

**Figure 1:**
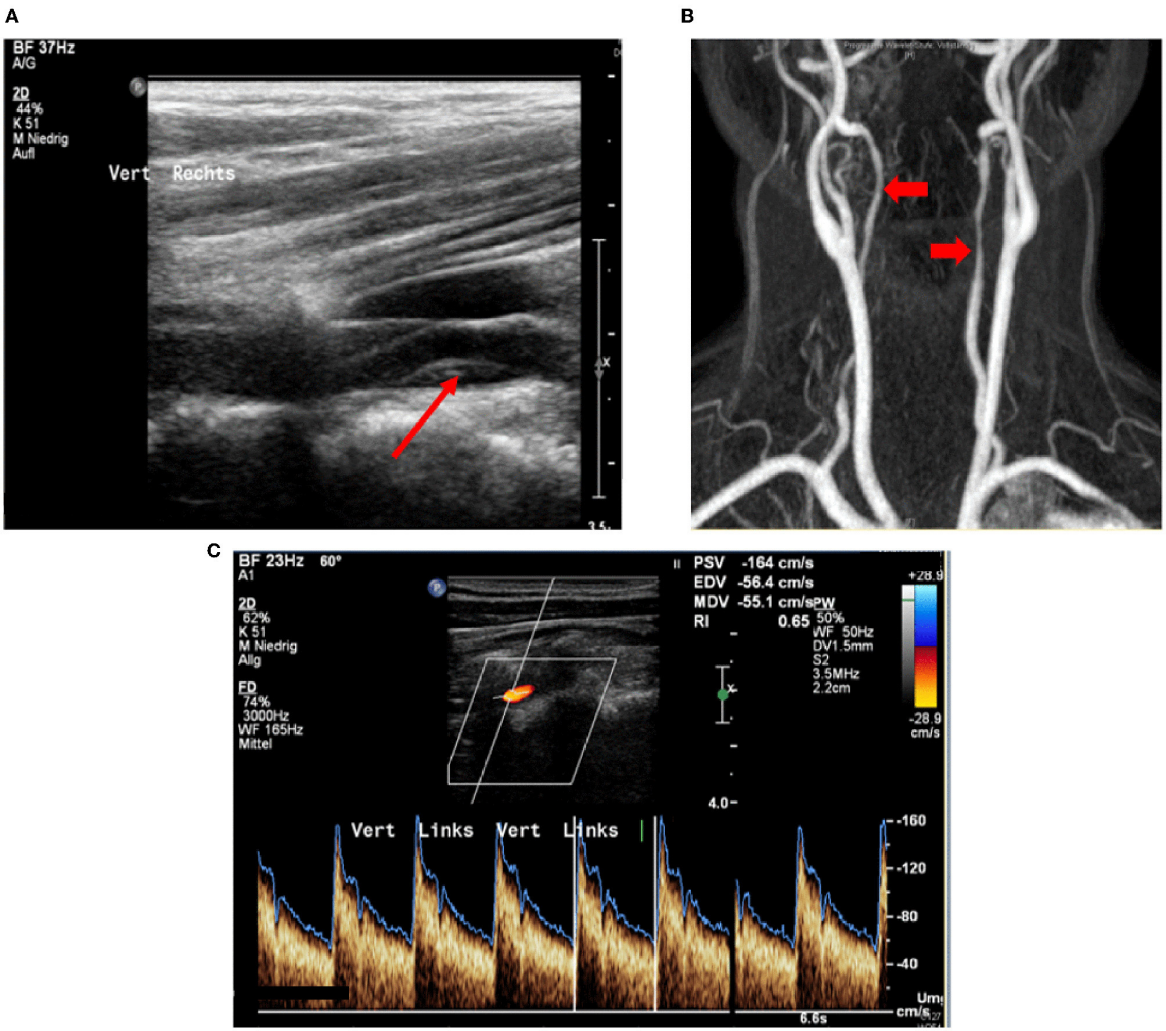
**(A)** 48-year-old patient with neck pain; wall hematoma in B-mode (indicated by red arrow). **(B)** Corresponding magnetic resonance angiography with evidence of long-distance, irregular lumen narrowing in both vertebral arteries (indicated by red arrows). **(C)** Wall hematoma and lumen narrowing in B-mode, moderate stenosis (blood flow velocity: 160 cm/s).

**Figure 2:**
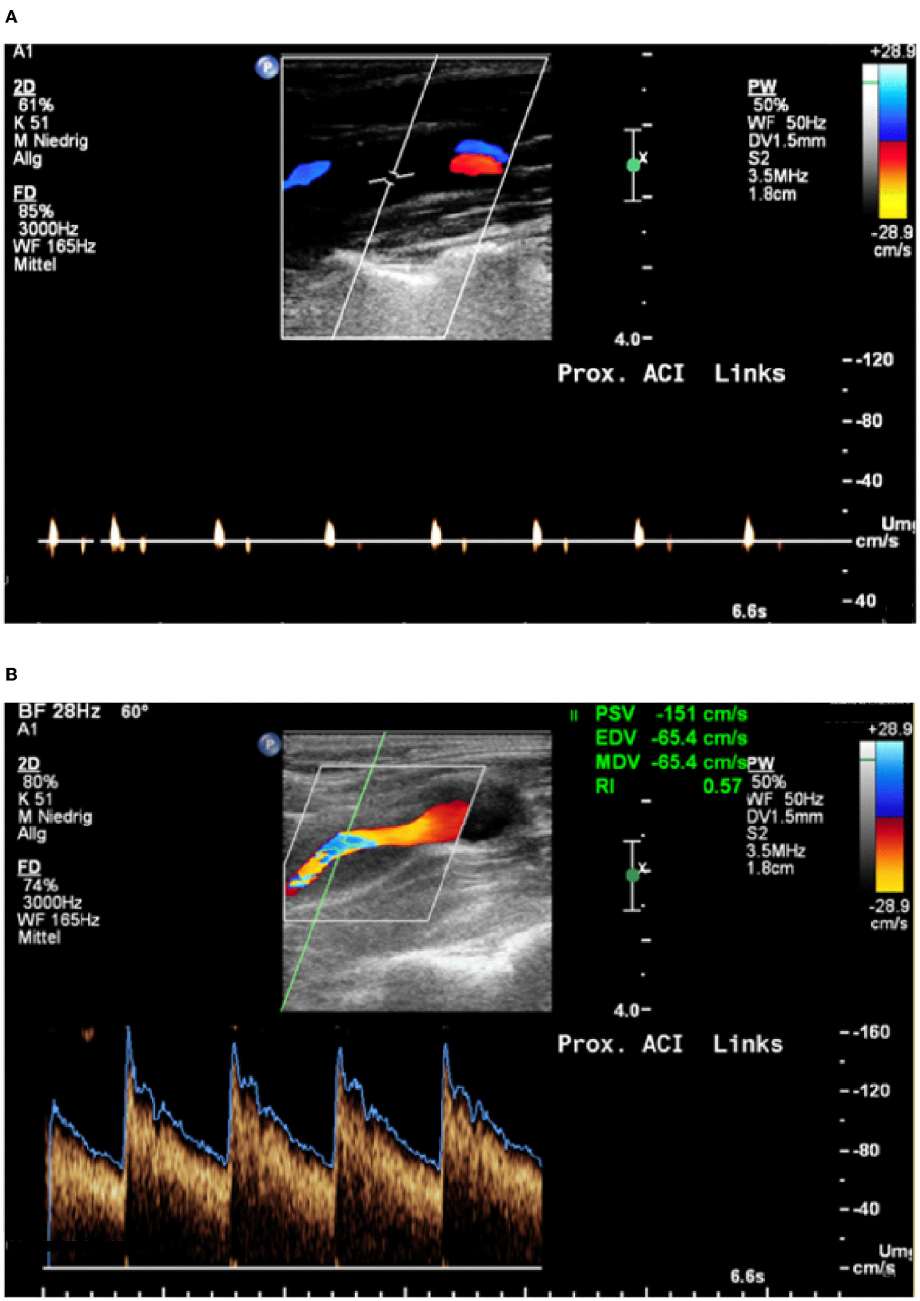
**(A)** 42-year-old patient with transient aphasia, paresthesia on the right, Horner syndrome on the left; Occlusion signal indicates occlusion of the internal carotid artery. **(B)** Neurovascular ultrasound done 3 days later shows recanalization with evidence of a wall hematoma.

In all patients, the brain supplying neck arteries were investigated by color-coded duplex sonography [linear array transducer: 7.5–12 MHz; Logiq 7 by General Electric [Boston (Massachusetts)], Epiq 5 by Philips (Hamburg (Germany))]. The color-coded duplex sonography included the examination of the common carotid arteries, the ICAs in their proximal and distal parts, the external carotid arteries, and the VAs in the segments V1, V2, V3, and V4. Duplex sonography was carried out by two specialized medical assistants with long-time experience under the supervision of a senior physician. The NASCET-criteria to measure the vascular stenosis in ultrasound are valid for assessing proximal segments close to the origin of the respective vessel in relation to distal vessel diameter. Given that stenoses due to sCAD are not necessarily located in the proximal part of a dissected vessel, but can just as well be located in the distal sections, we relied on the following classification, which can be applied to proximal and distal stenoses alike: (0) No visible stenosis in ultrasound, (1) stenosis without hemodynamic relevance, (2) stenosis with at least 50% difference in flow and/or acceleration of flow in comparison between the healthy and the affected sides, (3) 60–80% stenosis, (4) more than 80% stenosis, and finally, (5) arterial occlusion due to sCAD. These degrees of stenosis were attributed to each affected artery and follow the general classification of vascular stenosis by means of ultrasound. Figures 1, 2 show typical findings in neurovascular ultrasound and MRA in patients with sCAD.

## Results

A total of 259 sCADs were diagnosed in 224 patients. Only one ICA in 133 patients (59.4%), one VA in 58 patients (25.9%), and multiple arteries in 33 patients (14.7%) were affected (see Table 1). Overall, 74 patients (28.6%) had no visible stenosis in ultrasound, 18 (6.9%) had a stenosis without hemodynamic relevance, 23 (8.9%) had a stenosis with at least 50% difference in flow and/or acceleration of flow in comparison between the healthy and the affected sides, 25 (9.7%) showed 60–80% stenosis, 43 (16.6%) had more than 80% stenosis, and 76 patients (29.3%) had initial arterial occlusion due to sCAD.

**Table 1:** (Para-) clinical characteristics of study population and duration of follow-up.

	**All patients (*n* = 224)**	**No recurring dissections (*n* = 212)**	**Recurring dissections (*n* = 12)**	***P*-Value**	Improvement^*^of initial stenosis (*n* = 93^**^)	No improvement^*^of initial stenosis (*n* = 90^**^)	***P*-Value**
**Demographics**							
Age, mean (SD), y	45.9 ± 10.4	46 ± 10.4	41 ± 7.7	0.07	44.9 ± 11.1	46 ± 9.6	0.48
Male *n* (%)	129 (57.6)	124 (58.5)	5 (41.7)	0.29	48 (51.6)	52 (57.8)	0.41
**Comorbidities**, ***n*** **(%)**							
Hypertension	77 (34.4)	77 (36.3)	0	<0.01^†^	30 (31.6)	30 (33.3)	0.8
Diabetes mellitus	4 (1.8)	4 (1.9)	0	0.03^†^	2 (2.1)	2 (2.2)	0.96
Hypercholesterolemia	35 (15.6)	34 (16)	1 (8.3)	0.45	14 (14.7)	13 (14.4)	0.93
Atrial fibrillation	4 (1.8)	4 (1.9)	0	0.04^†^	2 (2.1)	3 (3.3)	0.64
Smoking	21 (9.4)	20 (9.4)	1 (8.3)	0.95	10 (10.5)	9 (10)	0.89
**Localization of dissection**, ***n*** **(%)**							
Internal carotid artery	133 (59.4)	133 (62.7)	0	<0.01^†^	46 (49.5)	58 (64.4)	0.49
Vertebral artery	58 (25.9)	58 (27.4)	0	<0.01^†^	18 (19.4)	29 (32.2)	0.49
Multiple arteries^***^	33 (14.7)	21 (9.9)	12 (100)	<0.01^†^	29 (31.2)	3 (3.3)	<0.01^†^
**Initial stenosis**, ***n*** **(%)**							
No visible stenosis	74 (28.6)	69 (30.1)	5 (16.7)	0.08	0 (0)	49 (52.7)	<0.01^†^
Stenosis without hemodynamic relevance	18 (6.9)	14 (6.1)	4 (13.3)	0.28	11 (9.4)	5 (5.4)	0.26
≥50% difference in flow and/or acceleration of flow comparing healthy and affected side	23 (8.9)	17 (7.4)	6 (20)	0.11	18 (15.4)	4 (4.3)	<0.01^†^
60–80% stenosis	25 (9.7)	16 (7.0)	9 (30)	0.01^†^	20 (17.1)	1 (1.1)	<0.01^†^
>80% stenosis	43 (16.6)	41 (17.9)	2 (6.7)	0.04^†^	34 (29.1)	5 (5.4)	<0.01^†^
Arterial occlusion	76 (29.3)	72 (31.4)	4 (13.3)	0.01^†^	34 (29.1)	29 (31.2)	0.74
**Clinical presentation**, ***n*** **(%)**							
Stroke	125 (55.8)	120 (56.6)	5 (41.7)	0.35	48 (51.6)	55 (61.1)	0.07
Transient ischemic attack	30 (13.4)	28 (13.2)	2 (16.7)	0.78	18 (19.4)	8 (8.9)	0.06
Local symptoms only	65 (29.0)	60 (28.3)	5 (41.7)	0.41	27 (29)	26 (28.9)	0.98
Initial NIHSS, mean ± SD	4.6 ± 6.8	5 ± 6.9	2 ± 3.5	0.02^†^	4.2 ± 7.2	4.2 ± 5.8	0.98
mRS at discharge, mean ± SD	1.2 ± 1.5	1.2 ± 1.5	0.7 ± 1.3	0.23	1.0 ± 1.4	1.1 ± 1.5	0.57
Favorable outcome (mRS ≤ 1), *n* (%)	161 (71.9)	153 (72.2)	10 (83.3)	0.34	73 (78.5)	64 (71.1)	0.25
Time between onset of symptoms and hospital admission [days], mean ± SD	4.5 ± 10.3	4.0 ± 10.5	4.0 ± 5.8	0.91	4.6 ± 10.5	4.7 ± 11.3	0.91
Follow-up duration [months], mean ± SD	15.3 ± 21	14.7 ± 20.9	27.2 ± 20.7	0.08	21.5 ± 27.6	17.4 ± 22.4	0.27

* *Patients were regarded as cases with improvement when at least one vessel showed a decreasing degree of stenosis; Only data on vessels with improvement are shown*.

** *Only patients with follow-up examinations were included;*

*** *Numbers represent cases, not single vessels. Consequently, the number of dissected vessels exceeds the number of affected patients. Patients with stenting and follow-up included (n = 18)*.

† *Statistically significant, p < 0.05.*

*SD, standard deviation; y, years; mRS, modified Rankin Scale; NIHSS, National Institutes of Health Stroke Scale*.

**Figure 3:**
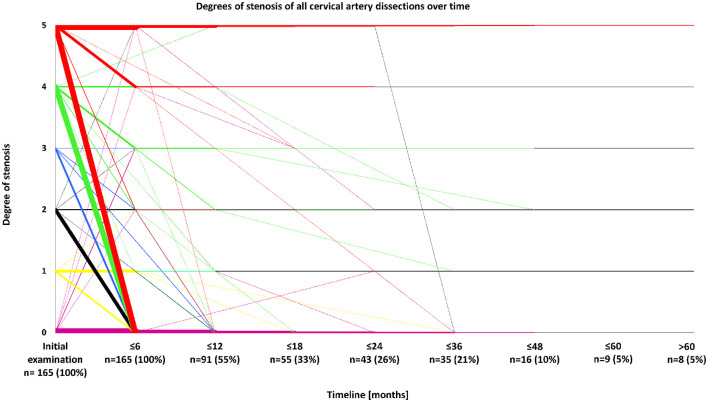
Degrees of stenosis of all cervical artery dissections over time. Degrees of stenosis were classified by neurovascular ultrasound according to the following scheme: 0: No visible stenosis, 1: Stenosis without hemodynamic relevance, 2: ≥50% difference in flow and/or acceleration of flow comparing healthy and affected sides, 3: 60–80% stenosis, 4: >80% stenosis, and 5: Arterial occlusion. N-numbers refer to patients being followed up for a certain period of time.

Sonographical follow-up was available in 183 patients, and comprised a time period of 21.5 ± 27.6 (patients with improving stenoses) or 17.4 ± 22.4 months (patients with constant or deteriorating stenoses) (mean ± standard deviation). Follow-up comprises a decreasing number of patients over time so that most data stem from the first year after disease onset, whereas only 5% of all patients were followed-up for a period of time of 5 years or more. Of 145 arteries with hemodynamically relevant stenosis due to sCAD (i.e., at least 50% difference in flow and/or acceleration of flow in comparison between the healthy and affected sides) being followed-up at least once, 90 (62.1%) showed an improvement of stenosis during the first 12 months after disease onset (Figure 3). Stenoses without hemodynamic relevance occurred in 65 arteries with follow-up examinations. Fifty-one of these arteries (78.5%) showed improving stenoses or already had no visible stenosis.

With regard to the total study population, 93 (51%) of 183 patients and 117 (55.7%) of 210 arteries had decreasing vascular stenosis during the entire follow-up-period. Of 32 individuals with multiple sCADs, 29 (90.6%) had an improvement of stenosis over time, whereas an increasing degree of stenosis was found in three cases (9.4%, *p* < 0.01). Twenty-seven out of 32 patients (84.4%) with multiple sCADs and follow-up examinations had a hemodynamically relevant arterial stenosis in one or more of the affected arteries. In 23 out of the aforementioned 27 patients (85.2%), at least one of the formerly hemodynamically relevant stenosed arteries did not have a hemodynamically relevant stenosis any more at time of last follow-up. In patients with single lesions due to sCAD, improvement was only seen in 64 out of 151 patients (42.4%). When only one ICA was affected (*n* = 104), improvement of arterial stenosis occurred in 46 patients (44.2%). Improvement of single vertebral artery lesions took place in 18 individuals (38.3%). Occluded vessels improved in 34 patients (54.0%) and stayed occluded in 29 patients (46.0%, *p* = 0.74). Improvement almost exclusively took place within the first year of follow-up. The most frequent course, especially in lesions with a hemodynamically relevant stenosis, was an improvement of initial stenosis, which predominantly occurred within the first 6–12 months after disease onset (see Figure 3). On the other hand, merely 11 patients (6.0%) had deteriorating degrees of stenosis over time (see Figure 3). Demographic characteristics including age, gender, and cerebrovascular risk factors did not differ between patients showing improvement or lacking improvement of initial degree of stenosis.

In patients without recurring dissections (*n* = 212), when compared to those with recurring dissections (*n* = 12), the lesions led to a more severe initial clinical presentation, measured by the National Institutes of Health Stroke Scale (NIHSS) at the time of hospital admission [5 ± 6.9 (sCAD without recurrence), 2 ± 3.5 (sCAD with recurrence)], whether or not stroke was diagnosed. Patients with recurring (27.2 ± 20.7 months) or improving stenoses (21.5 ± 27.6 months) were followed-up for the longest periods of time. Among twelve patients with recurring dissections, we observed early recurrence of sCAD as defined by Baracchini et al. in seven patients (58.3%), affecting an artery other than the initially affected one within a 2-week period after onset of symptoms, i.e., during the respective hospital stay (11). Five patients (41.7%) with late recurrence after hospital treatment, which tends to affect the initially affected vessel, were identified (11). Mean time for late recurrence was 80 days, median was 59 days, minimum duration was 22 days, and maximum duration was 174 days. Patients with recurring sCAD had significantly less cardiovascular risk factors, but the small number of affected individuals hinders comparison with non-recurring cases. Pseudoaneurysms, which bear an increased risk of arterio-arterial embolism, were found in three patients (25%) with and 16 patients (7.5%) without recurring dissection.

Stroke occurred in 125 patients (55.8%), local symptoms in 65 patients (29.0%) including Horner's syndrome, neck pain, or headache, and transitory ischemic attack occurred in 30 patients (13.4%) (Table 1). Clinical presentation of sCAD was comparable in all subgroups, i.e., patients with and without recurrence of sCAD or improvement of initial degree of stenosis.

Overall, 23 patients (10.3%) received endovascular stenting of the dissected vessel due to hemodynamically relevant stenosis. Post-interventional ultrasound showed no visible stenosis in the respective vessels. During follow-up, in-stent stenosis was detected in one patient, 8 months after initial interventional treatment. Despite a stroke as primary clinical presentation, he recovered well and modified Rankin Scale (mRS) score at discharge was 1. There were no recurrent stenoses in the remaining stented vessels. Patients who underwent endovascular stenting were not included in the analysis of arterial stenosis over time (see Figure 3). Overall, 161 patients (71.9%) had a good outcome, defined as an mRS score of zero or one, while the mean mRS score at discharge for all patients was 1.2 ± 1.5. One patient died. Outcome measures did not differ significantly between patients with or without improvement and recurrence of sCAD (see Table 1).

## Discussion

We here analyzed data of 224 patients with sCAD, diagnosed by CT/MRI and followed-up by neurovascular ultrasound. Mean number of total ultrasound examinations was 2.75 during a mean follow-up-period of 15.3 ± 21 months. Our analysis yielded the following main findings: The most frequent course, especially in sCAD-caused hemodynamically relevant stenosis with at least 50% difference in flow and/or acceleration of flow comparing healthy and affected sides or higher degrees of stenosis, was an improvement of initial stenosis which, predominantly, occurred within the first six to twelve months after disease onset. Of those patients with multiple sCADs, 90.6% had a decreasing degree of stenosis over time. In contrast, in patients with single lesions due to sCAD, improvement was only seen in 64 out of 151 patients (42.4%). Demographics and cerebrovascular risk factors did not differ considerably between patients with or without recurrence or improvement. Overall, recurrence of sCAD was a rare event during the follow-up-period (*n* = 12), and a less severe clinical course was evident [NIHSS at hospital admission (5 ± 6.9 (sCAD without recurrence), 2 ± 3.5 (sCAD with recurrence)]. Patients with recurring sCAD had significantly less cardiovascular risk factors, but the small number of affected individuals hinders comparison with non-recurring cases.

The distribution of affected arteries is in line with previous results, presented by Baracchini et al. (11) and von Babo et al. (12). We observed early recurrence of sCAD, affecting an artery other than the initially affected one, within a 2-week period after onset of symptoms in seven patients (58.3% of all patients with recurrent sCAD) compared to scarcer late recurrence, which tends to affect the initially affected artery too. Other authors suggested different pathologies underlying early and late recurrence in this context (11). Our findings also confirm the results of the following studies with regard to the period of time in which most changes in stenoses caused by sCAD occur. Steinke et al. described an overall recanalization rate of 68% in 50 sCADs of ICA after an average interval of 51 days (4). Caso et al. reported results from 38 patients with 19 sCADs of the ICA and 19 sCADs of the VA (5). Recanalization rate amounted to 42% without significant differences between both vascular territories. Sengelhoff et al. showed improvement or stability in the vast majority of arteries affected by sCAD (3). The highest rate of improvement, not only in occluded arteries, took place in the first 6 months (3). Thereafter, much less improvement could be detected. Finally, Shibahara et al. reported VA dissection primarily occurring within 6 months after onset (13). In comparison with other studies, our cohort comprises a higher number (*n* = 33) and percentage (14.7%) of multiple sCADs [(11): *n* = 4; 5.3%].

As far as therapy is concerned, most patients were exclusively treated with platelet inhibition or vitamin-K-antagonists for at least 6 months. These differences in therapeutic regimens might account for different outcomes and are closely linked to the retrospective design of our study. Given the good outcomes of treatment, measured on the mRS, most sCADs can safely be conservatively managed, with a majority of patients recovering well. This finding is in line with the observations published by Rao et al. (14).

Our study has strengths and limitations. A strength is the large patient cohort with a long follow-up-period up to 60 months. The retrospective design is a limitation and might have caused a certain bias regarding clinical and sonographic outcomes, given the lack of a scheduled time frame of the ultrasound investigations and different therapeutic strategies. Finally, a considerable number of patients were not examined by means of ultrasound for the entire follow-up period of 5 years. This fact is particularly due to either satisfactory regression of arterial stenosis at the last follow-up or a stable clinical and sonographic state during the last follow-up examinations.

## Conclusion

Decreasing degrees of stenosis and recanalization of occluded arteries were found in half of all patients, mainly within the first year. Recurrent sCAD was a rare event. In conclusion, the sonographical course of sCAD is highly dynamic within the first year after disease onset and should be monitored carefully. Patients with non-recurring sCAD seem to have a more severe disease onset.

## Data Availability Statement

The datasets presented in this article are not readily available because they contain personalized data underlying the final analysis and therefore cannot be shared. Requests to access the datasets should be directed to Jens Minnerup, Jens.Minnerup@ukmuenster.de.

## Author Contributions

DS extracted data and wrote the manuscript. WS and HW extracted data and contributed to writing the manuscript. RD and JM conceptualized and contributed to writing the manuscript. All authors contributed to the article and approved the submitted version.

## Conflict of Interest

HW is a member of the following scientific advisory boards/steering committees: Biogen, Sanofi Genzyme, MedDay Pharmaceuticals, Merck Serono, Novartis, and Roche. HW has received speaker honoraria and travel support from Alexion, Biogen, Cognomed, Evgen, Sanofi Genzyme, Impulze, KWHC, Merck Serono, Novartis, PeerVoice, Pennside, and PSL Group. HW has received compensation as a consultant from AbbVie, Actelion, Biogen, Sanofi Genzyme, Novartis, and Roche. HW has received research support from Biogen, Sanofi Genzyme, GlaxoSmithKline, Roche, and Solace Pharmaceuticals UK. JM has received grants from Deutsche Forschungsgemeinschaft, Bundesministerium für Bildung und Forschung BMBF, Else Kröner-Fresenius-Stiftung, EVER Pharma Jena GmbH, and Ferrer International, travel grants from Boehringer Ingelheim, and speaking fees from Bayer Vital. The remaining authors declare that the research was conducted in the absence of any commercial or financial relationships that could be construed as a potential conflict of interest.

## Publisher's Note

All claims expressed in this article are solely those of the authors and do not necessarily represent those of their affiliated organizations, or those of the publisher, the editors and the reviewers. Any product that may be evaluated in this article, or claim that may be made by its manufacturer, is not guaranteed or endorsed by the publisher.
